# Evolution of genome space occupation in ferns: linking genome diversity and species richness

**DOI:** 10.1093/aob/mcab094

**Published:** 2021-07-14

**Authors:** Tao Fujiwara, Hongmei Liu, Esteban I Meza-Torres, Rita E Morero, Alvaro J Vega, Zhenlong Liang, Atsushi Ebihara, Ilia J Leitch, Harald Schneider

**Affiliations:** Center for Integrative Conservation, Xishuangbanna Tropical Botanical Garden, Chinese Academy of Sciences, Yunnan, China; Makino Herbarium, Tokyo Metropolitan University, 1-1 Minami-osawa, Hachioji, Tokyo, Japan; Xishuangbanna Tropical Botanical Garden, Chinese Academy of Sciences, Yunnan, China; Instituto de Botánica del Nordeste, Universidad Nacional del Nordeste, Consejo Nacional de Investigaciones Científicas y Técnicas, Corrientes, Argentina; Instituto Multidiscipinario de Biologia Vegetal, Universidad Nacional de Cordoba, Consejo Nacional de Investigaciones Científicas y Tecnicas, Cordoba, Argentina; Instituto de Botánica del Nordeste, Universidad Nacional del Nordeste, Consejo Nacional de Investigaciones Científicas y Técnicas, Corrientes, Argentina; Center for Integrative Conservation, Xishuangbanna Tropical Botanical Garden, Chinese Academy of Sciences, Yunnan, China; Department of Botany, National Museum of Nature and Sciences, Tsukuba, Japan; Royal Botanic Gardens, Kew, London, UK; Center for Integrative Conservation, Xishuangbanna Tropical Botanical Garden, Chinese Academy of Sciences, Yunnan, China

**Keywords:** DNA C-values, genome evolution, genome size, macroevolution, polyploidy, pteridophytes

## Abstract

**Background and Aims:**

The dynamics of genome evolution caused by whole genome duplications and other processes are hypothesized to shape the diversification of plants and thus contribute to the astonishing variation in species richness among the main lineages of land plants. Ferns, the second most species-rich lineage of land plants, are highly suitable to test this hypothesis because of several unique features that distinguish fern genomes from those of seed plants. In this study, we tested the hypothesis that genome diversity and disparity shape fern species diversity by recording several parameters related to genome size and chromosome number.

**Methods:**

We conducted *de novo* measurement of DNA C-values across the fern phylogeny to reconstruct the phylogenetic history of the genome space occupation in ferns by integrating genomic parameters such as genome size, chromosome number and average DNA amount per chromosome into a time-scaled phylogenetic framework. Using phylogenetic generalized least square methods, we determined correlations between chromosome number and genome size, species diversity and evolutionary rates of their transformation.

**Key Results:**

The measurements of DNA C-values for 233 species more than doubled the taxon coverage from ~2.2 % in previous studies to 5.3 % of extant diversity. The dataset not only documented substantial differences in the accumulation of genomic diversity and disparity among the major lineages of ferns but also supported the predicted correlation between species diversity and the dynamics of genome evolution.

**Conclusions:**

Our results demonstrated substantial genome disparity among different groups of ferns and supported the prediction that alterations of reproductive modes alter trends of genome evolution. Finally, we recovered evidence for a close link between the dynamics of genome evolution and species diversity in ferns for the first time.

## INTRODUCTION

Increasing evidence demonstrates a close correlation between the evolvability – the innate capacity of a lineage to evolve by adapting to changes in environmental conditions (Kirchner and Gerhart, 1998; Pigliucci, 2008; Rabosky *et al.*, 2013) – and the dynamics of genome evolution (Leitch and Leitch, 2012; Bromham *et al.*, 2015; Puttick *et al.*, 2015) with the latter shaped by processes such as whole genome duplications (WGD), amplification of repetitive DNA (RDA) and diploidization involving DNA deletions (DPD) (Freeling *et al.*, 2015; Soltis *et al.*, 2015; Schubert and Vu, 2016). These processes have been considered not only to cause the ‘C-value enigma’ – a 64 000-fold genome size disparity in eukaryotes and 2400-fold disparity in plants – but also to contribute to the remarkable differences in species richness among major lineages of eukaryotes and land plants (Gregory, 2004; Leitch and Leitch, 2013; Slijepcevic, 2018; Choi *et al.*, 2020). Indeed, these arguments have been discussed as a consequence of observed differences among land plant lineages in their genome diversity as indicated by holoploid genome size, chromosome numbers and composition of genomic components such as non-coding DNA elements (Leitch and Leitch, 2012; Pellicer *et al.*, 2018). Whereas studies focused so far on angiosperms and gymnosperms have recovered substantial differences in genome diversity, less attention has been given to ferns, despite their genomes being characterized by several unique features.

Containing ~11 000 extant species (PPGI, 2016), ferns are not only the second most species-rich lineage of vascular plants, but are characterized by (1) the highest frequency of polyploidy-enforced speciation events (Wood *et al.*, 2009), (2) accumulation of large chromosome numbers including the largest number of chromosomes recorded among all organisms (Khandelwal, 1990; Clark *et al.*, 2016), (3) accumulation of medium to large genome sizes including one of the largest genomes recorded (Hidalgo *et al.*, 2017; Pellicer *et al.*, 2018), (4) a positive correlation between genome size and chromosome number and long terminal repeat-retrotransposon (LTR-RT) insertion time (Nakazato *et al.*, 2008; Barker, 2013; Baniaga and Barker, 2019), and (5) a high rate of recurrent WGD during the phylogenetic history of several fern lineages (Clark *et al.*, 2016; Huang *et al.*, 2020). Lastly, the first complete and partially sequenced fern genomes recovered evidence for recurrent WGD and spread of repeat elements in homosporous ferns (Wolf *et al.*, 2015; Marchant *et al.*, 2019), whereas the heterosporous ferns were distinct in their relatively small genome size and composition of non-coding DNA (Li *et al.*, 2018). These results were consistent with the predictions made by previous investigations using different types of evidence to explore fern genomes (Barker and Wolf, 2010; Barker, 2013; Haufler, 2014; Clark *et al.*, 2016). Due to the unique characteristics observed in fern genomes and their key phylogenetic position as the sister lineage of seed plants, an exploration of fern genome space has been expected to shed new light on genome evolution in land plants (Rensing, 2017). Additionally, ferns show not only a diversity of life histories (Petersen and Burd, 2017), adaptation to low-light conditions (Schneider *et al.*, 2004) and ecological adaptive strategies (Schuettpelz and Pryer, 2009), but also a highly uneven phylogenetic distribution of extant species diversity in which 78 % of extant fern diversity belongs to only three lineages in Polypodiales (PPGI, 2016). Together, these characteristics make ferns a highly suitable group to test the association between genome diversity and lineage diversification.

In this study, we reconstructed evolutionary patterns of genome size across the whole fern phylogeny with the aim of exploring the hypothesis that genome diversity and disparity shape fern species diversity. Specifically, the experimental setup was designed to expand the phylogenetic and taxonomic coverage of DNA C-values as required to explore the fern genome space occupation using five parameters: holoploid genome size (1C), gametic chromosome number (*n*), monoploid genome size (1C*x*), basic chromosome number (*x*) and average DNA amount per chromosome (1C/*n*). The last value enabled us to quantify the packaging of DNA in the form of chromosomes. In general, the fern genome has been considered to be very stable based on the observation of a positive correlation between chromosome number and genome size, which was interpreted as a consequence of recurrent WGD combined with conservation of chromosome structure (CCS) and delayed diploidization (Nakazato *et al.*, 2008; Barker, 2013; Clark *et al.*, 2016). This correlation was confirmed here using the highly improved dataset. In turn, we determined the phylogenetic pattern of observed genome size variation because lineage-specific traits and historical events were expected to contribute to the accumulated genome disparity of ferns. This prediction is supported by the observations on genome size variation among lineages of ferns (Dyer *et al.*, 2013; Clark *et al.*, 2016; Liu *et al.*, 2019) and the reported differences between the genomes of heterosporous ferns and their homosporous relatives (Klekowski and Baker, 1966; Haufler, 2014; Li *et al.*, 2018). Finally, the hypothesis that genome diversity shapes fern species diversity was tested by specifically analysing link between species richness and the dynamics of genome evolution estimated as a rate of genome size evolution.

## MATERIALS AND METHODS

### Taxon sampling

DNA C-values were obtained with the aim of at least doubling the number of species with reported genome sizes and the coverage in particular lineages lacking data until now. The latest fern classification (PPGI, 2016) was employed as a guide to obtain species estimates and a robust phylogenetic framework. New genome size measurements were generated from material collected mostly in southern China and southern South America by combining fieldwork and utilizing of living collections at the Xishuangbanna Tropical Botanical Garden, CAS (China) and Wuhan Botanical Garden, CAS (China), and the Tsukuba Botanical Garden (Japan). This expanded sampling enabled us to overcome the bias towards species occurring in temperate regions of Europe and North America that have hampered previous analyses (e.g. Clark *et al.*, 2016).

### De novo *measurement of genome size and chromosome counts*

DNA C-values were obtained using flow cytometry with propidium iodide following preparation procedures as described previously (Clark *et al.*, 2016). Leaf fragments of the studied taxon were chopped together with an internal standard – either *Glycine max* ‘Polanka’ (2C = 2.50 pg) (Doležel *et al.*, 1994), *Pisum sativum* ‘Ctirad’ (2C = 9.09 pg) (Doležel *et al.*, 1992) or *Vicia faba* ssp. *faba* var *equina* ‘Inovec’ (2C = 26.90 pg) (Doležel *et al.*, 1992) – in an isolation buffer – either General Purpose Buffer (Loureiro *et al.*, 2006) or Ebihara Buffer (Ebihara *et al.*, 2005). Fluorescence intensities were analysed on a BD FACSVerse (BD Bioscience, San Jose, CA, USA). Each taxon was measured at least three times using leaves from the same individual, if possible, to enable the calculation of mean 2C-values and standard deviations for each taxon based on the fluorescence ratios between the sample and internal standards. Genome size data obtained in this study are provided in Supplementary Data Table S1. New chromosome counts were carried out using established protocols (Takamiya, 1993; Table S2 and Figs S1–S4).

### Data extraction from databases and the literature

Besides the newly generated data, previously published DNA C-values were obtained from the ‘Pteridophyte DNA C-values database’ (https://cvalues.science.kew.org) (Leitch et al., 2019) and recent publications (Clark *et al.*, 2016; Dauphin *et al.*, 2016; Yahaya *et al.*, 2016; Fujiwara *et al*., 2017, 2018; Chang *et al.*, 2018; Li *et al.*, 2018; Kuo and Li, 2019; Supplementary Data Table S1). Chromosome counts were assembled from the ‘Index to Plant Chromosome Number’ (IPCN; http://www.tropicos.org/project/ipcn) (Goldblatt and Johnson, 1979) and the ‘Chromosome Count Database’ (CCDB; http://ccdb.tau.ac.il/; Rice *et al.*, 2015).

### Characterizing fern genomes

We analysed five genomic parameters: (1) holoploid genome size (1C), (2) gametic chromosome number (*n*), (3) basic chromosome number (*x*), (4) monoploid genome size (1C*x*) and (5) average DNA amount per chromosome (1C/*n*). Basic chromosome number (*x*) was determined as the lowest gametic chromosome number in each genus (Manton and Vida, 1968), whilst ploidy level for each taxon was estimated based on the basic chromosome number for the genus. Most fern genera show highly conserved chromosome numbers although some exhibit extensive chromosome number variation [e.g. *Hymenophyllum* (Hennequin *et al.*, 2010) and *Lepisorus* (Wang *et al.*, 2010)]. In such cases, we first inferred ploidy level for each taxon by comparing the chromosome number of the taxon with the gametic chromosome number reported for the group to which the taxon belongs. Subsequently, we estimated the basic chromosome number for the taxon by dividing its chromosome number by its ploidy level. Monoploid genome size (1C*x*) is defined as the genome size per basic chromosome number (Greilhuber *et al.*, 2005) and was obtained by dividing the 2C-value by ploidy level for each taxon. The average DNA amount per chromosome (1C/*n*) was calculated by dividing the 1C-value by the gametic chromosome number (*n*).

### Phylogenetic analysis

Total genomic DNA was extracted from silica gel-dried samples using the EasyPure Plant Genomic DNA Kit (Transgen Biotech, Beijing, China). We amplified the plastid gene *rbcL* using standard PCR protocols (Schuettpelz and Pryer, 2007). Sanger sequencing was outsourced to the BGI sequencing service (http://www.genomics.cn/en). We also downloaded *rbcL* and *atpB* sequences from GenBank. Of the 561 species with genome size data, 430 were selected based on the availability of DNA sequences and data for all genomic parameters examined in this study, for the phylogenetic analysis. All sequences, including *Selaginella helvetica* (AB574644), *Isoetes sinensis* (AB574660) and *Lycopodium clavatum* (AB574626) which were used as the outgroup, were merged into a single sequence matrix and aligned using MAFFT (Katoh and Standley, 2013), followed by manual editing in AliView (Larsson, 2014). The best model of nucleotide substitution was selected with jModelTest 2 (Darriba *et al.*, 2012). Tree reconstruction was performed using maximum likelihood (ML) as implemented in RAxML-HPC2 8.2.6 (Stamatakis, 2014) on the CIPRES Science Gateway portal (http://www.phylo.org/). We used GTRGAMMA as the substitution model and performed 1000 bootstrap (BS) replicates. The tree topology for the phylogenetic location of orders and families was constrained based on PPGI (2016). The obtained ML tree is shown in Supplementary Data Fig. S5. To obtain an ultrametric tree, we used the penalized likelihood method using PATHd8 (Smith and O’Meara, 2012). We used 17 fixed fossils and secondary calibration points in the best ML tree in RAxML based on fossil records and the estimated ages from a large-scale integrated fossil study reconstructing the divergence times of ferns (Testo and Sundue, 2016) (Table S3). The obtained ultrametric tree is shown in Fig. S6.

### Statistical analysis

Each genomic parameter was explored with Shapiro–Wilk normality tests and the R package qqplot. Because all of the parameters deviated significantly from a normal distribution (*P* < 0.001), they were log-transformed before subsequent analyses.

For the phylogenetic signal for each genomic parameter, Pagel’s λ was estimated using the fitContinuous function in the GEIGER package (Harmon *et al.*, 2008) in R. The significance of estimated values was tested by a likelihood ratio test (LRT) compared with the value in a lambda = 0 model. To detect the heterogeneity of genome size evolution, three a *priori* scenarios were tested under Brownian motion (BM) and Ornstein–Uhlenbeck (OU) processes using OUwie (Beaulieu *et al.*, 2012). The employed scenarios were organized from simple to increasingly complex models: (1) a scenario in which all ferns evolve under the same trend (Singular Model); (2) a scenario in which heterosporous ferns evolve under a distinct trend from homosporous ferns (Heterosporous Fern Model); and (3) a scenario in which all orders evolve independently under distinct trends from each other (All Orders Model). The BM process is a random walk process with a pure stochastic change to any value in trait space, regulated by only the rate parameter (σ^2^), while the OU process controls changes of value by incorporation of an attractor (α) that is the strength to move back to an optimum value (θ), together with σ^2^. For the OU process with multiple regimes, we applied four OU processes, OUM (only θ varies among distinct regimes), OUMV (θ and σ^2^), OUMA (θ and α) and OUMVA (θ, α and σ^2^) with different assumptions about which of the three parameters, σ^2^, α and θ, vary among distinct regimes. For these analyses, we prepared a multi-regimes phylogeny using the paintSubTree function in the phytools package (Revell, 2012). We conducted model selection for three genomic parameters – 1C, 1C*x* and 1C/*n* – and selected the best model by calculation of a sample size-corrected Akaike information criterion (AICc). To discover additional rate shifts for 1C, 1C*x* and 1C/*n*, we conducted l1ou (Khabbazian *et al.*, 2016) analysis, which is a computationally efficient approach that uses a lasso method to automatically detect evolutionary shifts under the OU process. We used the estimate_shift_configuration function in this package with pBIC model as the selection criterion. We calculated bootstrap values for each shift location with 500 iterations using the l1ou_bootstrap_support function. The same analyses were also conducted for a reduced dataset containing only Polypodiales.

We examined the relationships between genomic parameters, (1C vs. *n*, 1C vs. 1C/*n*) by employing phylogenetic generalized least square (PGLS) analysis (Grafen, 1989), as implemented by the pgls function with lambda = ‘ML’ in the caper package (Orme, 2013), against four different groups, ‘All ferns’ ‘Homosporous ferns’ ‘Leptosporagiate ferns’ and ‘Polypodiales.’ The same analytical settings were employed to examine the correlation between 1C*x* and *x* as necessary to detect a putative bias created by neo-polyploid taxa.

To examine the predicted correlations between total species number and evolutionary rates of genome size, PGLS analyses were performed under the same settings as described above. For this, we conducted order-level comparisons. However, species diversity in ferns is highly biased towards Polypodiales, which accounts for almost 80 % of extant species diversity in ferns (PPGI, 2016). To avoid the expected bias, the order Polypodiales was separated into families instead of a single unit. This treatment takes into account that species diversity is highly variable even within Polypodiales and its pattern of genome size evolution is also heterogeneous although other basal orders show more stable patterns (see Results and Discussion below). The evolutionary rates of 1C, 1C*x* and 1C/*n* for each order, and, for Polypodiales, each family that included genome size data for more than three species, were estimated as σ^2^ under Brownian motion calculated with the fitContinuous function in the GEIGER package (Supplementary Data Table S4). In parallel, we also examined correlations between diversification rates and evolutionary rates of genome size. We adopted a conservative way to estimate diversification rate in each clade by calculating the rate under a single constant-rate model based only on clade age and species richness (see Magallón and Sanderson, 2001). The diversification rates for each order, and each family of Polypodiales, were calculated using the bd.ms function in the GEIGER package with the parameters epsilon and missing set to zero. We used species richness and clade age for each group from PPGI (2016) and Testo and Sundue (2016), respectively. All values were log-transformed before analysis.

## RESULTS

### Genome size variation throughout ferns

Our *de novo* measurements from 233 species combined with previously published DNA C-values greatly increased the taxon coverage from ~2.2 % to ~5.3 %, including 100 % of the orders and 50 % of the fern genera (Table 1 and Supplementary Data Table S1). Taxon coverage varied across the fern phylogeny ranging from 2.8 % in Hymenophyllales to 80 % in Equisetales (Table 1). Ferns showed a mean value of 12 377 Mb in holoploid genome size (1C), varying 629.5-fold from 234 Mb for *Salvinia cucullata* to 147 291 Mb for *Tmesipteris obliqua*, with a mean of 8599 and 387.6-fold range in monoploid genome size (1C*x*) and a mean of 223 Mb and 108.7-fold range in average DNA amount per chromosome (1C/*n*).

**Table 1.** Summary of mean, minimum (Min), maximum (Max) size and size range of holoploid genome size (1C), monoploid genome size (1C*x*) and average DNA amount per chromosome (1C/*n*). Total species number SN is based on PPGI (2016).

Order	SN	SN-1C	TC-1C (%)	Mean 1C (Mb)	Min 1C (Mb)	Max 1C (Mb)	1C size range	Mean 1C*x* (Mb)	Min 1C*x* (Mb)	Max 1C*x* (Mb)	1C*x* size range	Mean 1C/*n* (Mb)	Min 1C/*n* (Mb)	Max 1C/*n* (Mb)	1C*/n* size range
**All ferns**	10 578	561	5.3	12 377	234	147 291	629.45	8599	95	36 823	387.61	223	10	1087	108.7
**Homosporous ferns**	10 388	552	5.3	12 501	1823	147 297	80.79	8695	1823	36 823	20.2	225	44	1087	24.7
**Equisetales**	15	12	80	22 517	11 066	31 833	2.88	22 516	11 066	31 833	2.88	208	102	294	2.88
**Ophioglossales**	112	23	20.5	23 140	7878	64 108	8.14	10 299	4231	14 455	3.42	220	73	321	4.4
**Psilotales**	17	3	17.6	87 919	60 117	147 291	2.45	34 752	30 053	36 823	1.23	668	577	708	1.23
**Marattiales**	111	12	10.8	9685	4430	20 548	4.64	7262	4425	10 748	2.43	183	113	269	2.38
**Osmundales**	18	9	50	15 337	13 158	20 547	1.56	15 337	13 158	20 547	1.56	697	598	933	1.56
**Hymenophyllales**	434	12	2.8	19 857	10 494	31 408	2.99	16 661	5247	25 236	4.81	559	146	1088	7.45
**Gleichniales**	172	13	7.6	3978	1824	18 934	10.38	2522	1824	3183	1.75	59	40	80	2
**Schizaeles**	190	11	5.8	12 594	5555	22 939	4.13	9120	4675	14 484	3.1	314	199	483	2.43
**Salviniales**	82	9	11	1405	235	2572	10.94	1064	96	1917	19.97	75	11	202	18.36
**Cyatheales**	713	23	3.2	10 257	2465	24 054	9.76	8473	2465	13 868	5.63	125	44	210	4.77
**Polypodiales**	8714	434	5	11 185	2380	59 164	24.86	7695	2308	32 758	14.19	211	61	798	13.08
Saccolomatineae	18	1	5.6	37 907	–	–	–	9477	–	–	–	205	–	–	–
Lindsaeineae	234	7	3	7464	3550	12 959	3.65	4841	3156	7540	2.39	105	66	151	2.29
Pteridiineae	1211	66	5.5	9989	2582	34 099	13.21	5912	2308	13 435	5.82	197	80	448	5.6
Dennstaedtiineae	265	12	4.5	7755	3166	14 802	4.68	4686	3166	8963	2.83	137	66	168	2.55
Aspleniineae	2775	147	5.3	9755	2380	26 558	11.16	6229	2380	19 746	8.3	170	61	581	9.52
Polypodiineae	4208	201	4.8	13 209	3702	59 164	15.98	10 003	3707	32 758	8.84	266	100	799	7.99

SN: species number, SN-1C: number of species with reported 1C genome size, TC-1C: taxon coverage in 1C genome size.

### Genome size evolution throughout the phylogeny of ferns

**Fig. 1.**
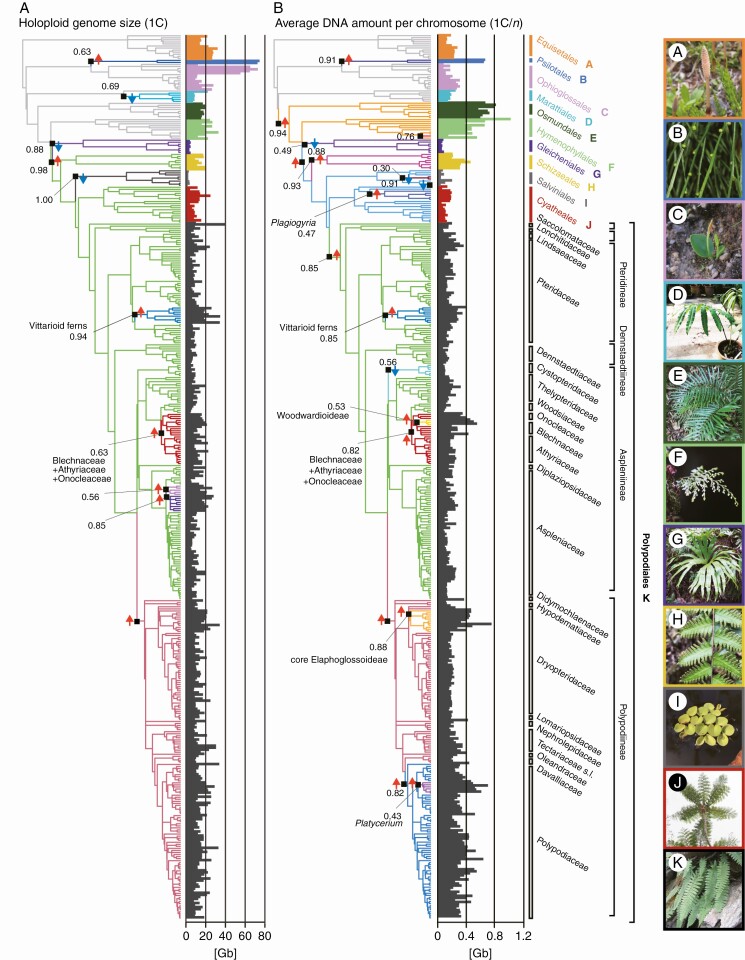
Phylogeny with clades coloured to reflect rate shifts under an Ornstein–Uhlenbeck process suggested by the 1lou method for holoploid genome size (1C) (A) and average DNA amount per chromosome (1C/*n*) (B). Black squares and numbers indicate the locations of rate shifts and bootstrap support values for these shift placements. Red and blue arrows near black squares indicate up and down rate shifts respectively. Bar plot located next to each phylogeny depicts the parameter value for each species with different colours indicating different orders. Each picture on the right showing a representative of each order. Classification according to PPGI (2016).

All genomic parameters examined showed significant phylogenetic signals (*P* < 0.001), specifically 1C: λ = 0.907, 1C*x*: λ = 0.954, 1C/*n*: λ = 0.948, *n*: λ = 0.797, *x*: λ = 1.00 (Supplementary Data Table S5). The OUwie-selected All-order model with OU processes was found to be the best fit model for the three genomic parameters (1C, 1C*x* and 1C/*n*) compared with the other models (Table 2). For each of the three genomic parameters, although the OUMA and OUMVA models showed much higher log likelihoods and lower AICc than any other models (Table S6), the parameter values estimated in the models showed large deviations from those in other models and thus these models were discarded due to their inappropriate model fitting. Therefore, among the OU processes, OUM (which infers only different optimum values among different groups) was selected as the best model for 1C-values and OUMV (which infers a different rate parameter and optimum values) was selected for 1C*x* and 1C/*n* values (Table 2; Table S6). The l1ou algorithm recovered several evolutionary shifts in genome size that occurred across the phylogeny for the three genomic parameters, showing high heterogeneity of genome evolution in ferns (Fig. 1; Fig. S7).

**Table 2.** Parameter estimates in the best models for 1C, 1C*x* and 1C/*n* from OUwie analysis. The optimum value (**θ**) in each genomic parameter was back-transformed from the logistic value.

Genomic parameter	1C			1C*x*			1C/*n*		
Best model	OUM			OUMV			OUMV		
Parameter	α	σ^2^	θ (Mb)	α	σ^2^	θ (Mb)	α	σ^2^	θ (Mb)
**Equisetales**	0.7869	0.7069	20039	0.7869	0.1592	20039	0.7869	0.1592	186
**Psilotales**	0.7869	0.7069	72127	0.7869	0.0003	36064	0.7869	0.0003	694
**Ophioglossales**	0.7869	0.7069	18639	0.7869	0.2938	8832	0.7869	0.4116	177
**Marattiales**	0.7869	0.7069	8157	0.7869	0.0489	7267	0.7869	0.0486	183
**Osmundales**	0.7869	0.7069	15862	0.7869	0.0241	15862	0.7869	0.0241	721
**Hymenophyllales**	0.7869	0.7069	19536	0.7869	0.2932	17079	0.7869	0.3836	501
**Gleicheniales**	0.7869	0.7069	2501	0.7869	0.0706	2501	0.7869	0.0774	56
**Schizaeales**	0.7869	0.7069	13198	0.7869	0.0625	9498	0.7869	0.1407	295
**Salviniales**	0.7869	0.7069	887	0.7869	1.05	711	0.7869	1.1006	51
**Cyatheales**	0.7869	0.7069	8240	0.7869	0.3673	7634	0.7869	0.2935	116
**Polypodiales**	0.7869	0.7069	10400	0.7869	0.415	7746	0.7869	0.4064	215

### Correlation among genome size and chromosome number

**Fig. 2.**
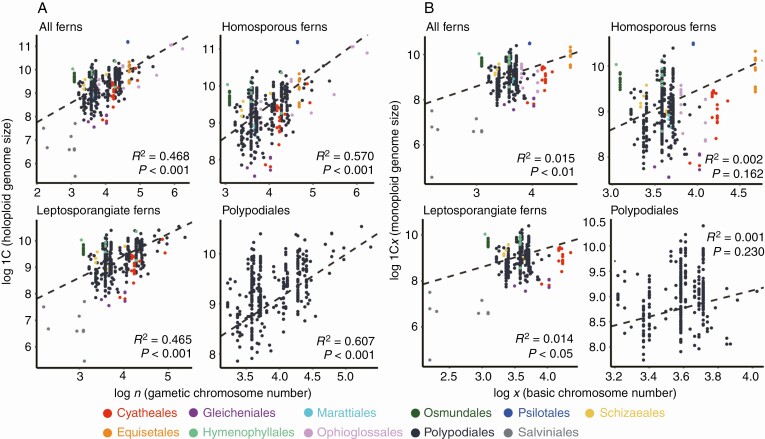
Scatter plot showing the correlation between holoploid genome size (1C) and gametic chromosome number (*n*) (A) and the correlation between monoploid genome size (1C*x*) and basic chromosome number (*x*) (B) for ‘All ferns,’ ‘Homosporous ferns’, ‘Leptosporangiate ferns’ and ‘Polypodiales’. Each dot corresponds to one taxon with its color indicating the order to which it belongs according to the legend. Dashed lines indicate the regression lines calculated using PGLS. Classification according to PPGI (2016).

Holoploid genome size (1C) was significantly positively correlated with gametic chromosome number (*n*) and average DNA amount per chromosome (1C/*n*) across the phylogeny of ferns, and in the major groups tested independently (Fig. 2A; Supplementary Data Table S7 and Fig. S8). In each group tested, the chromosome number fitted better with the holoploid genome size than the 1C/*n* value, as indicated by λ > 0.900 and *R*^2^ > 0.45. With exclusion of the bias created by neo-polyploidy, monoploid genome size (1C*x*) was significantly correlated with basic chromosome number for all ferns and leptosporangiate ferns (Fig. 2B). However, this correlation was not supported for homosporous ferns and Polypodiales (Fig. 2B; and Table S7).

### The rate of genome size evolution is correlated with species diversity

**Fig. 3.**
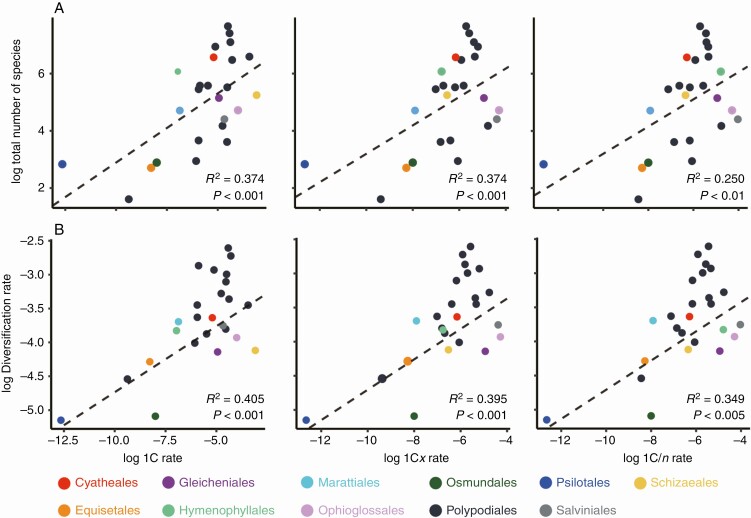
Scatter plots showing correlations between the total number of species (A) and diversification rate (B), with the evolutionary rate of three genomic parameters, holoploid genome size (1C) (left), monoploid genome size (1C*x*) (middle) and average DNA amount per chromosome (1C/*n*) (right). Classification according to PPGI (2016). Each dot corresponds to the mean value for an order or, for Polypodiales, each family, with its color indicating the order to which it belongs according to the legend. Solid and dashed lines indicate the regression lines calculated for ‘All ferns’ and ‘Homosporous ferns’ using PGLS.

Significant positive correlations were observed between the total number of species and the evolutionary rates of each of the three genomic parameters: 1C rate, 1C*x* rate and 1C/*n* rate (Fig. 3A; Supplementary Data Table S8). Similar positive correlations were also identified between diversification rates and rates of genome size evolution (Fig. 3B). In contrast, there was no significant correlation between the total number of species and the mean values for the three genomic parameters (Fig. S9 and Table S9).

## DISCUSSION

The newly generated DNA C-values of 233 fern species greatly improved taxon coverage to 5.3 %, including 100 % of the orders and 50 % of the fern genera according to PPGI (2016). This coverage is more than double that of previous summaries, 2.3 % and 2.2 % in Clark *et al.* (2016) and Pellicer *et al.* (2018), respectively, and is the best among land plant lineages containing more than 2000 species (Pellicer *et al.*, 2018). Using our updated dataset, we examine genome size disparity throughout the fern phylogeny and its association with species diversification.

### Genome size disparity across the phylogeny of ferns

The three genomic parameters analysed to explore genome space evolution – 1C, 1C*x* and 1C/*n* – show significantly high phylogenetic signals throughout the fern phylogeny (Supplementary Data Table S5), suggesting genome space disparity among the main lineages. Consistent with this, OUwie analyses selected the All order model with OU process (where each order has a distinct evolutionary trend from each other) as the best model for all three genomic parameters, specifically the OUM model for 1C, and the OUMV model for 1C*x* and 1C/*n* (Table 2; Table S6). Because the OU process better fits to all genomic parameters for genome size than the BM process, we conclude that each fern order has evolved distinct optimal values of genome size and structure (Table 2). The selection of OUMV mode – which assumes not only an optimal value but also a rate parameter as varying between distinct groups – as the best model for 1C*x* and 1C/*n* suggests that different fern orders display differences in the dynamics of monoploid genome size and chromosome size evolution. The ancient sister lineages Ophioglossales and Psilotales share the accumulation of large genomes in 1C but show opposite trends in 1C/*n* (Table 1), where they show contrasting rates of chromosome size evolution and optimal values (694 Mb for Psilotales and 177 Mb for Ophioglossales, Table 2). Among homosporous ferns, Gleicheniales is distinct by its relatively small genomes that are the consequence of rate shifts towards genome size reduction (Tables 1 and 2; Fig. 1), and its 1C/*n* values are comparable to those of heterosporous ferns. On the other hand, other ancient lineages of leptosporangiate ferns, namely Osmundales and Hymenophyllales, evolved towards one of the largest optimal values of 1C/*n* among all ferns (721 Mb for Osmundales and 501 Mb for Hymenophyllales, Table 2), consequently developing large 1C and 1C*x* values (Table 1). However, the two lineages show contrasting rates of genome size evolution in 1C*x* and 1C/*n*. Whereas Osmundales exhibits the second slowest rate of genome size evolution (see Schneider *et al.*, 2015), Hymenophyllales shows relatively faster genome change, consistent with the report of basic chromosome number variations (Hennequin *et al.*, 2010). The largest optimal values of 1C/*n* were consistent with previous reports documenting very large chromosomes in at least some of these ferns (Lovis, 1978). In contrast to other orders of ferns, the Polypodiales exhibits heterogeneous trends of genome size evolution as a result of rate shifts in genome size evolution within this order. Almost half of the evolutionary shifts in genome size are located within the order Polypodiales (5/10 shifts in 1C, 10/21 in 1C*x* and 9/18 in 1C/*n*; see Fig. 1 and Supplementary Data Fig. S7). The frequency of shifts is consistent with the genome disparity recovered by the largest ranges of 1C and the second largest of 1C*x* and 1C/*n* among all ferns (Table 1). Most notably, the three most species-rich fern lineages (PPGI, 2016), namely Aspleniineae, Polypodiineae and Pteridineae, contributing together ~78 % of the extant fern diversity, are highly variable in the three genomic parameters (1C, 1C*x* and 1C/*n*) used to elucidate the genome disparity.

Genome size disparity is not restricted to homosporous ferns but also occurs between homosporous and heterosporous ferns. Heterosporous ferns are distinct in their trend towards the smallest values in all three core parameters (1C, 1C*x* and 1C/*n*) and the largest disparity and evolutionary rate of 1C*x* and 1C/*n* detected among ferns (Table 1). This result is consistent with the conclusions from the whole genome sequences of these ferns (Li *et al.*, 2018). The rapid transformation of the genomes of these ferns was probably caused by the transition from homosporous to heterosporous reproduction. Thus, the observed result supports the hypothesis that changes in the reproductive system accelerated the rate of genome evolution in this relatively young lineage of ferns (Haufler, 2014). However, it should be noted that these results for 1C*x* and 1C/*n* may be confused as a consequence of misassignment of chromosome numbers and ploidy level for species in this group. For example, although the reported genome sizes of *Salvinia cuculata* and *S. molesta* are 0.48 and 4.45 pg/2C respectively, the chromosome numbers and ploidy level for both species were reported to be 2*n* = 45 and pentaploid (Tatuno and Takei, 1969). Thus, some Salviniales species may show intraspecific chromosome variation that may cause the ambiguous link between genome size and chromosome number. Therefore, the link between genome size and chromosome number in Salviniales requires further exploration to elucidate genome size disparity between homosporous and heterosporous ferns.

### Repeated WGD with delayed DPD and CCS

Holoploid genome size (1C) was significantly positively correlated with both chromosome number (*n*) and average DNA amount per chromosome (1C/*n*) across the phylogeny of ferns, including the major groups tested independently (Fig. 2A; Supplementary Data Table S7 and Fig. S8). The model with chromosome number fitted better with 1C than that with 1C/*n* for all groups examined (Table S7). This result was consistent with previous reports showing that ferns are one of two lineages of land plants showing this correlation (Nakazato *et al.*, 2008; Barker, 2013; Clark *et al.*, 2016). This pattern may be explained by repeated WGD in the phylogeny of ferns combined with delayed genome size reduction (such as DPD) as reflected by the conservation of chromosome structure (CCS). The latter was elucidated using the 1C/*n* value. This conclusion was also supported by the observation of a conservation of LTRs since their insertion (Baniaga and Barker, 2019) and the spread of repeat elements in homosporous fern genomes (Marchant *et al.*, 2019).

1C*x* was significantly positively correlated with basic chromosome number (*x*) across all ferns (*P* < 0.01) and leptosporangiate ferns (*P* < 0.05), but this correlation was weak as indicated by the *R*^2^ values (*R*^2^ = 0.015 for all ferns and *R*^2^ = 0.014 for leptosporangiate ferns) and rejected for homosporous ferns (*P* = 0.162) and Polypodiales (*P* = 0.230) (Fig. 2B; Supplementary Data Table S7). This result was consistent with recently published results showing that 1C/*n* is not as constant as previously assumed, despite a general trend towards conservation of relatively small chromosomes in most fern lineages compared to angiosperms and gymnosperms (Dyer *et al.*, 2013; Liu *et al.*, 2019). Due to the high frequency of neopolyploidy combined with the trend to conserve chromosome structure, the contribution of processes such as DNA deletion and selective DNA amplification was elucidated by focusing only on monoploid genome size. Nonetheless, our analysis against a reduced dataset containing only Polypodiales found additional evidence supporting the breakdown of CCS in this most speciose fern lineage (Fig. S10), where several shifts in 1C/*n* value largely contributed to contraction and expansion of genome size. This conclusion is supported by the relatively small numbers of reported dysploid chromosome series in ferns (Lovis, 1978; Hennequin *et al.*, 2010; Wang *et al.*, 2010). These results suggest that deviation of CCS is more common in species-rich lineages compared to the overall patterns recovered in ferns.

### Genome size diversity and species richness

The total number of species and diversification rate were correlated with the rate of evolution for the three genomic parameters – 1C, 1C*x* and 1C/*n* (Fig. 3; Supplementary Data Table S8), but not with their mean values (Fig. S9 and Table S9). This result matches the reported correlation between the dynamics of genome evolution and diversification rates in angiosperms (Puttick *et al.*, 2015). In ferns, the correlation may be mainly explained by the high frequency of polyploidy (Wood *et al.*, 2009) because lineages showing high rates of 1C evolution are known to include many polyploid species such as Ophioglossales (Khandelwal, 1990; Dauphin *et al.*, 2018), Hymenophyllales (Ebihara *et al.*, 2005; Nitta *et al.*, 2011) and most of the families in Polypodiales, such as Aspleniaceae (Schneider *et al.,* 2017). However, polyploidy does not explain the observed patterns alone, because we also found a correlation between species richness and the evolutionary rate of 1C*x* and 1C/*n*. These findings suggest that other processes such as DPD and RDA may have contributed to fern diversification, although their impact has been substantially lower than in angiosperms (Schubert and Vu, 2016). In particular, the species-rich lineages of Polypodiales showed evidence for enhanced rates of genome evolution as a consequence of contributions from WGD, RDA and DPD, and leading to enhanced diversification rates that contributed to the evolutionary success of this fern lineage. This result supports recurrent WGD during the diversification of derived ferns as suggested from transcriptome data (One Thousand Plant Transcriptomes Initiative, 2019; Huang *et al.*, 2020). In turn, the less dynamic genome space explored by ferns may also explain their rather small species diversity compared to angiosperms.

## CONCLUSION

Our results provide strong support for the hypothesis that the diversification of plant lineages has been shaped by the dynamics of their genome evolution. Instead of focusing on whole genome sequences or transcriptomes, parameters linked to the amount of DNA and its packaging in chromosomes were used to elucidate the dynamics of genome space evolution in ferns. This approach has the disadvantage that we cannot trace changes in genome composition such as the contribution to genome evolution of different kinds of repetitive DNA or the fate of duplicated genes arising from ancient whole genome duplications. However, this disadvantage has to be taken in the context of the much denser and more balanced taxon sampling achieved than in any study using sequenced genomes alone (Li *et al.*, 2018; One Thousand Plant Transcriptomes Initiative, 2019). Future studies will hopefully integrate genomic and transcriptomic sequence evidence into the framework created using the parameters used in this study. Despite the limited genomic sequence data available for ferns, our results are highly consistent with studies that have used complete or partially sequenced genomes of heterosporous and homosporous ferns (Wolf *et al.*, 2015; Li *et al.*, 2018). Furthermore, we have confirmed several predictions based on the hypothesis that different trends in chromosome and genome evolution between homosporous and heterosporous ferns could be attributed to their reproductive system, as proposed in previous studies on fern genetics (Klekowski and Baker, 1966; Haufler, 2014).

Expanding on our findings, the remarkable difference in species richness among land plants may be explained by differences in the dynamics of genome evolution. The remarkable success of angiosperms – exceeding all other land plant lineages in their species richness, phenotypic diversity and ecological importance – is arguably the consequence of innovations enabling much faster rates of genome evolution compared to their sister lineage the gymnosperms, and their more distant relatives, ferns and bryophytes (Leitch and Leitch, 2012; Puttick *et al.*, 2015; Pellicer *et al.*, 2018). In turn, lower rates of genome evolution may explain the lower species numbers of ferns. However, the enhanced rate of genome evolution shown here, may also have promoted the success of some recently diverging lineages. WGDs alone fail to explain these successes despite their prominence because other process, such as the amplification and purging of repetitive DNA, and diploidization with DNA deletion, may have crucially contributed to unleash polyploidy-driven innovations.

## SUPPLEMENTARY DATA

Supplementary data are available online at https://academic.oup.com/aob and consist of the following. Table S1: Summary of the information on genome size obtained and used for the present study. Table S2: Plant materials used for chromosome counting in this study with their chromosome counts. Table S3: Summary of the fossil calibration points used in this study. Table S4: Total number of species and rate values estimated under the Brownian motion model for 1C, 1C*x* and 1C/*n* in each order and each family in Polypodiales. Table S5: Pagel’s lambda for each genomic parameter, holoploid genome size, monoploid genome size, average DNA amount per chromosome, gametic chromosome number and basic chromosome number examined in this study. Table S6: Summary comparison between singular and multiple models, and Brownian motion and Ornstein–Uhlenbeck processes. Table S7: PGLS statistics of the correlation between holoploid genome size and related parameters such as chromosome number and average DNA amount per chromosome plus the correlation between monoploid genome size and basic chromosome number calculated for ‘all ferns’, “homosporous ferns,’ ‘leptosporangiate ferns’ and ‘Polypodiales’. Table S8: PGLS statistics of the relationships between each of total number of species and diversification rate, and evolutionary rates of the three genomic parameters, holoploid genome size, monoploid genome size and average DNA amount per chromosome. Table S9: PGLS statistics of the relationships between each of total number of species and diversification rate, and the mean values for the three genomic parameters, holoploid genome size, monoploid genome size and average DNA amount per chromosome. Figs S1–S4: Mitotic metaphase chromosomes observed in this study. Fig. S5: ML tree obtained in this study. Fig. S6: Ultrametric tree used in this study. Fig. S7: Phylogeny with clades coloured to reflect rate shifts in Ornstein–Uhlenbeck parameters suggested by the 1lou method for monoploid genome size. Fig. S8: Scatter plot showing the relationships between holoploid genome size and average DNA amount per chromosome for ‘All ferns,’ ‘Homosporous ferns,’ ‘Leptosporangiate ferns’ and ‘Polypodiales.’ Fig. S9: Scatter plot showing the correlation between each of total number of species and diversification rate, and mean values for the three genomic parameters, holoploid genome size, monoploid genome size and average DNA amount per chromosome. Fig. S10: Polypodiales phylogeny with clades coloured to reflect rate shifts in Ornstein–Uhlenbeck parameters suggested by the l1ou method for holoploid genome size, and average DNA amount per chromosome.

mcab094_suppl_Supplementary_S01

mcab094_suppl_Supplementary_S02
