# Supplementary material for: Evolution of genome space occupation in ferns: linking genome diversity and species richness
Source: Ann Bot. 2021 Jul 14;131(1):59–70. doi: 10.1093/aob/mcab094 (PMC9904345; doi:10.1093/aob/mcab094)
Supplement: mcab094_suppl_Supplementary_S02 [file mcab094_suppl_supplementary_s02.doc]

**Electronic supplementary material**

**Evolution of genome space occupation in ferns: linking genome diversity and species richness.**

Tao Fujiwara, Hongmei Liu, Esteban I. Meza-Torres, Rita E. Morero, Alvaro J. Vega, Zhenlong Liang, Atsushi Ebihara, Ilia J. Leitch and Harald Schneider

**Table S2** **Plant materials used in this study with their chromosome counts.**

Each species name in bold indicates species whose chromosome counts was reported for first time in this study.

| **Family** | **Taxon** | **Chromosome number** | **Voucher** | **Figure** |
| --- | --- | --- | --- | --- |
| Lindsaeaceae | *Lindsaea javanensis Blume* | 2n = 188 | T. Fujiwara 20190413-4 (HITBC) | Fig. S1-1 |
| Pteridaceae | ***Aleuritopteris subvilosa* (Hooker) Ching** | **2n = 116** | LHM2183 (HITBC) | Fig. S1-2 |
|  | *Cheilanthes tenuifolia* (N. L. Burman) Swartz | 2n = 116 | T. Fujiwara 20181230-1 (HITBC) | Fig. S1-3 |
|  | ***Coniogramme emeiensis* Ching & K. H. Shing** | **2n = 120** | MEG-18Y-37 (HITBC) | Fig. S1-4 |
|  | *Coniogramme fraxinea* (D. Don) Fée ex Diels | 2n = 60 | T. Fujiwara 20181122-5 (HITBC) | Fig. S1-5 |
|  | ***Coniogramme suprapilosa* Ching** | **2n = 120** | MEG-18Y-175 (HITBC) | Fig. S1-6 |
|  | ***Haplopteris forrestiana* (Ching) E.H. Crane** | **2n = 120** | MEG-18Y-141 (HITBC) | Fig. S1-7 |
|  | *Pteris kawabatae* Sa. Kurata | 2n = 58 | MEG-18Y-140 (HITBC) | Fig. S1-8 |
| Aspleniaceae | ***Asplenium delavayi* (Franchet) Copeland** | **2n = 72** | LHM2194 (HITBC) | Fig. S1-9 |
|  | *Asplenium pseudolaserpitiifolium* Ching | 2n = 144 | MEG-18Y-3 (HITBC) | Fig. S2-10 |
| Hypodematiaceae | *Hypodematium crenatum* (Forsskål) Kuhn & Decken | 2n = 164 | T. Fujiwara 20190528-1 (HITBC) | Fig. S 2-11 |
| Dryopteridaceae | *Dryopteris stenolepis* (Baker) C. Christensen | 2n = ca. 123 | T. Fujiwara 29181113-14 (HITBC) | Fig. S2-12 |
|  | *Bolbitis heteroclita* (C. Presl) Ching | 2n = 82 | T. Fujiwara 20181030-13 (HITBC) | Fig. S2-13 |
|  | ***Lomagramma matthewii* (Ching) Holttum** | **2n = 82** | T. Fujiwara 20181113-6 (HITBC) | Fig. S2-14 |
| Tectariaceae | *Tectaria fauriei* Tagawa | **2n = 164** | T. Fujiwara 20181030-6 (HITBC) | Fig. S2-15 |
| Polypodiaceae | ***Lepisorus henryi*  (Hieronymus ex C. Christensen) Li Wang** | **2n = 144** | PE-21 (HITBC) | Fig. S2-16 |
|  | ***Lepisorus obscurevenulosus* (Hayata) Ching** | **2n = 50** | MEG-18Y-136 (HITBC) | Fig. S3-17 |
|  | ***Lepisorus pseudonudus* Ching** | **2n = 52** | MEG-18Y-91 (HITBC) | Fig. S3-18 |
|  | ***Lepisorus sordidus* (C. Christensen) Ching** | **2n = 50** | FRG-201812-17 (HITBC) | Fig. S3-19 |
|  | *Lepisorus aff. tosaensis* (Makino) H. Itô | 2n = ca. 150 | MEG-18Y-132 (HITBC) | Fig. S3-20 |
|  | *Leptochilus pteropus*  (Blume) Fraser-Jenk. | 2n = 144 | MEG-18H-40 (HITBC) | Fig. S3-21 |
|  | ***Loxogramme cuspidate* (Zenker) M. G. Price** | **2n = ca. 70** | FRG-201901-193 (HITBC) | Fig. S3-22 |
|  | *Microsorum cuspidatum* (D.Don) Tagawa | 2n = 72 | MEG-18Y-128 (HITBC) | Fig. S3-23 |
|  | *Microsorum insigne* (Blume) Copeland | 2n = 72 | T. Fujiwara 20181030-5 (HITBC) | Fig. S3-24 |
|  | ***Pyrrosia drakeana*  (Franchet) Ching** | **2n = 74** | MEG-18Y-179 (HITBC) | Fig. S4-25 |
|  | ***Pyrrosia tonkinensis* (Giesenhagen) Ching,** | **2n = 148** | LHM2176 (HITBC) | Fig. S4-26 |

**Table S3** **Summary list for calibrating point used in this study.**

| **Group** | **Age (Ma)** | **Fossil** | **Reference** |
| --- | --- | --- | --- |
| Lycophyte crown | min 392 max 451 | †*Leclercquia complexa* | Wellman et al. (2009) |
| All ferns crown | min 385 max 451 | †*Ibyka amphikoma* | Skog and Banks (1973) |
| Equistales | min 151 | 2nd calibration | Testo and Sundue (2016) |
| Ophioglossales | min 205.32 | 2nd calibration | Testo and Sundue (2016) |
| Psilotales | min 66.31 | 2nd calibration | Testo and Sundue (2016) |
| Marratiales | min 162.04 | 2nd calibration | Testo and Sundue (2016) |
| Osmundales | min 203.74 | 2nd calibration | Testo and Sundue (2016) |
| Hymenophyllales | min 242.93 | 2nd calibration | Testo and Sundue (2016) |
| Gleicheniales | min 278.13 | 2nd calibration | Testo and Sundue (2016) |
| Schizeales | min 298.27 | 2nd calibration | Testo and Sundue (2016) |
| Salviniales | min 154.38 | 2nd calibration | Testo and Sundue (2016) |
| Cyatheales | min 219.23 | 2nd calibration | Testo and Sundue (2016) |
| Polypodiales | min 59.96 | 2nd calibration | Testo and Sundue (2016) |
| Cheilanthoideae clown | min 98 | †*Heinrichsia cheilanthoides* | Regalado et al. (2019) |
| Thelypteridaceae stem | min 98 | †*Holttumopteris burmensis* | Regalado et al. (2018) |
| Lindsaeaceae clown | min 98 | †Lindsaeaceae | Regalado et al. (2017) |
| Monachosorum stem | min 98 | †*Krameropteris resinatus* | Schneider et al. (2016) |

**Table S4 Total number of species (SN) and rate values estimated under brownian motion (BM) model for 1C, 1Cx and 1C/n in each order and each family in Polypodiale.**

| **Order/Family** | **SN** | **1C rate** | **1C*x* rate** | **1C/n rate** |
| --- | --- | --- | --- | --- |
| Ophioglossales | 112 | 0.01825742 | 0.01370105 | 0.01372804 |
| Salviniales | 82 | 0.009345174 | 0.01244261 | 0.01777718 |
| Davalliaceae | 65 | 0.008530511 | 0.008498244 | 0.008530511 |
| Gleicheniales | 172 | 0.007143296 | 0.007143296 | 0.007124834 |
| Thelypteridaceae | 1034 | 0.005979641 | 0.005525506 | 0.004786586 |
| Aspleniaceae | 730 | 0.03199348 | 0.004833484 | 0.004830459 |
| Pteridaceae | 1211 | 0.01253279 | 0.004649421 | 0.004407993 |
| Polypodiaceae | 1652 | 0.0120451 | 0.003790075 | 0.004322097 |
| Dryopteridaceae | 2115 | 0.01111953 | 0.003327832 | 0.003327838 |
| Blechnaceae | 265 | 0.002805303 | 0.002986806 | 0.003821835 |
| Athyriaceae | 650 | 0.01369661 | 0.002700007 | 0.00269102 |
| Nephrolepidaceae | 19 | 0.002327061 | 0.002327061 | 0.002327061 |
| Cyatheales | 713 | 0.005510165 | 0.00211798 | 0.001853244 |
| Tectariaceae | 250 | 0.01076524 | 0.002065535 | 0.002114686 |
| Woodsiaceae | 39 | 0.002626705 | 0.0016888 | 0.002104403 |
| Schizaeales | 190 | 0.04559487 | 0.001465047 | 0.001745303 |
| Dennstaedtiaceae | 265 | 0.004158213 | 0.001248682 | 0.00132745 |
| Hymenophyllales | 434 | 0.000947039 | 0.001158863 | 0.008346176 |
| Cystopteridaceae | 37 | 0.01065698 | 0.001107271 | 0.001053196 |
| Lindsaeaceae | 234 | 0.002634868 | 0.000897365 | 0.000812058 |
| Marattiales | 111 | 0.001042678 | 0.000369313 | 0.000365239 |
| Osmundales | 18 | 0.000335062 | 0.000335062 | 0.000335062 |
| Equisetales | 15 | 0.000254329 | 0.000254329 | 0.000254329 |
| Onocleaceae | 5 | 8.47E-05 | 8.47E-05 | 0.000216257 |
| Psilotales | 17 | 3.22E-06 | 3.22E-06 | 3.22E-06 |
| Polypodiales | 8714 | 0.013575 | 0.003562 | 0.003692 |

**Table S5 Pagel’s lambda for each trait examined in this study. P value indicates if Pagel’s lambda value for a given trait is significantly different from lambda = 0. Significant P values are printed in bold.**

| **Trait** | **Pagel’s lambda** | **P value** |
| --- | --- | --- |
| 1C | 0.9069 | **P < 0.001** |
| 1C*x* | 0.9538 | **P < 0.001** |
| 1C/n | 0.9484 | **P < 0.001** |
| n | 0.7967 | **P < 0.001** |
| *x* | 0.9995 | **P < 0.001** |

**Table S6. Summary of Model comparison between singular and multiple models, and Brownian Motion (BM) and Ornstein-Uhlenbeck (OU) process. In the Singular model, all ferns evolve under the same parameters. In the Heterosporous model, only heterosporous ferns (Salviniales) deviate from other ferns. In All orders, model all eleven orders evolve under distinct parameters from each other. The model with bold is the best model for each trait.**

| **Trait** | **Model** | | **lnL** | **AICc** | **delta AICc** |
| --- | --- | --- | --- | --- | --- |
| **1C** | BM1 | Singular model | -1096.131 | 2196.289 | 1406.8555 |
|  | BMS | Heterosporous fern model | -1095.29 | 2196.636 | 1407.2025 |
|  | BMS | All orders model | -935.2116 | 1895.168 | 1105.7345 |
|  | OU1 | Singular model | -510.7813 | 1027.619 | 238.1855 |
|  | OUM | Heterosporous fern model | -469.6246 | 947.3429 | 157.9094 |
|  | **OUM** | **All orders model** | **-381.2814** | **789.4335** | 0 |
|  | OUMV | Heterosporous fern model | -395.0518 | 838.8095 | 49.376 |
|  | OUMV | All orders model | -395.0518 | 838.8095 | 49.376 |
|  | OUMA | Heterosporous fern model | 486.1139 | -962.087 | 1751.5205 |
|  | OUMA | All orders model | 2.33E+14 | -4.66E+14 | 4.66115E+14 |
|  | OUMVA | Heterosporous fern model | 756.8848 | -1501.572 | 2291.0055 |
|  | OUMVA | All orders model | 15022014902 | -30044029738 | 30044030527 |
| **1C*x*** | BM1 | Singular model | -667.9009 | 1339.83 | 750.327 |
|  | BMS | Heterosporous fern model | -663.1672 | 1332.391 | 742.888 |
|  | BMS | All orders model | -561.4833 | 1147.711 | 558.208 |
|  | OU1 | Singular model | -422.4242 | 850.9046 | 261.4016 |
|  | OUM | Heterosporous fern model | -360.2726 | 728.6389 | 139.1359 |
|  | OUM | All orders model | -299.9173 | 626.7055 | 37.2025 |
|  | OUMV | Heterosporous fern model | -358.8729 | 727.8866 | 138.3836 |
|  | **OUMV** | **All orders model** | **-270.3986** | **589.503** | 0 |
|  | OUMA | Heterosporous fern model | 1180.805 | -2351.469 | 2940.972 |
|  | OUMA | All orders model | 1868872585 | -3737745121 | 3737745711 |
|  | OUMVA | Heterosporous fern model | 476.2785 | -940.3594 | 1529.8624 |
|  | OUMVA | All orders model | 36896.62 | -73721.6 | 74311.103 |

**Table S6. Continued**

| **Trait** | **Model** | **grouping** | **lnL** | **AICc** | **delta AICc** |
| --- | --- | --- | --- | --- | --- |
| **1C/n** | BM1 | Singular model | -690.9466 | 1385.921 | 793.0166 |
|  | BMS | Heterosporous fern model | -687.0556 | 1380.167 | 787.2626 |
|  | BMS | All orders model | -583.6579 | 1192.06 | 599.1556 |
|  | OU1 | Singular model | -394.5204 | 795.0968 | 202.1924 |
|  | OUM | Heterosporous fern model | -372.3674 | 752.8284 | 159.924 |
|  | OUM | All orders model | -298.9585 | 624.7877 | 31.8833 |
|  | OUMV | Heterosporous fern model | -371.0069 | 752.1547 | 159.2503 |
|  | **OUMV** | **All orders model** | **-272.0993** | **592.9044** | 0 |
|  | OUMA | Heterosporous fern model | 1486.656 | -2963.171 | 3556.0754 |
|  | OUMA | All orders model | 1.37E+15 | -2.75E+15 | 2.74836E+15 |
|  | OUMVA | Heterosporous fern model | 911.6289 | -1811.06 | 2403.9644 |
|  | OUMVA | All orders model | 39047.48 | -78028.97 | 78621.8744 |

**Table S7 PGLS statistics of the correlation between holoploid genome size (1C) and related parameters such as chromosome number (n) and average chromosome length (1C/n) plus the correlation between monoploid genome size (1C*x*) and basic chromosome number (*x*) calculated for “all ferns”, "homosporous ferns", “leptosporangiate ferns”, “Polypodiales”, related to Figure 2. The values given include 𝜆-value slope value, p-value, adjusted R^2^, adjusted p-value, and degree of freedom (D.F.). P-values < 0.05 are printed in bold. For 𝜆, the mean value is given plus the minimum and maximum confidence value.**

| **Group** | **Response** | **Predictor** | **λ (95% CI)** | **Slope** | **adj. *R^2^*** | ***p* -value** | **D.F.** |
| --- | --- | --- | --- | --- | --- | --- | --- |
| **All ferns** | 1C | n | 0.949 (0.925, 0.965) | 0.83667 | 0.4678 | ***p* < 0.001** | 430 |
|  | 1C | 1C/n | 0.831(0.715, 0.867) | 0.83016 | 0.4143 | ***p* < 0.001** | 430 |
|  | 1C*x* | *x* | 0.950 (0.926, 0.966) | 0.54138 | 0.0153 | ***p* < 0.01** | 430 |
| **Homosporous ferns** | 1C | n | 0.978 (0.964, 0.987) | 0.85752 | 0.5703 | ***p* < 0.001** | 422 |
|  | 1C | 1C/n | 0.767 (0.658, 0.847) | 0.81800 | 0.3873 | ***p* < 0.001** | 422 |
|  | 1C*x* | *x* | 0.977 (0.963, 0.986) | 0.33392 | 0.0023 | *p* = 0.162 | 422 |
| **Leptosporangiate ferns** | 1C | n | 0.947 (0.921, 0.965) | 0.827416 | 0.4645 | ***p* < 0.001** | 397 |
|  | 1C | 1C/n | 0.739 (0.610, 0.834) | 0.832478 | 0.4300 | ***p* < 0.001** | 397 |
|  | 1C*x* | *x* | 0.948 (0.922, 0.965) | 0.5324 | 0.0137 | ***p* < 0.05** | 397 |
| **Polypodiales** | 1C | n | 0.971 (0.950, 0.985) | 0.86567 | 0.6072 | ***p* < 0.001** | 338 |
|  | 1C | 1C/n | 0.647 (0.434, 0.790) | 0.82885 | 0.3657 | ***p* < 0.001** | 338 |
|  | 1C*x* | *x* | 0.970 (0.948, 0.984) | 3.53E-01 | 0.0013 | *p* = 0.230 | 338 |

**Table S8 PGLS statistics of the relationships between each of species number and diversification rate, and evolutionary rate of three traits, holoploid genome size (1C), monoploid genome size (1C*x*) and average DNA amount per chromosome (1C/n). P-values < 0.05 are printed in bold. For 𝜆, the mean value is given plus the minimum and maximum confidence value.**

| **Response** | **Predictor** | **λ (95% CI)** | **Slope** | **adj. *R^2^*** | **P-value** | **D.F.** |
| --- | --- | --- | --- | --- | --- | --- |
| total number of species | 1C rate | 0.000 (NA, 0.413) | 0.48295 | 0.3735 | ***p* < 0.001** | 23 |
|  | 1C*x* rate | 0.000 (NA, 0.412) | 0.49666 | 0.2780 | ***p* < 0.001** | 23 |
|  | 1C/n rate | 0.000 (NA, 0.386) | 0.47593 | 0.2498 | ***p* < 0.01** | 23 |
| Diversification rate | 1C rate | 0.493 (NA, 0.955) | 0.19493 | 0.4047 | ***p* < 0.001** | 23 |
|  | 1C*x* rate | 0.479 (NA, 0.906) | 0.22274 | 0.3950 | ***p* < 0.001** | 23 |
|  | 1C/n rate | 0.447 (NA, 0.865) | 0.21379 | 0.3491 | ***p* < 0.005** | 23 |

**Table S9 PGLS statistics of the relationships between each of total number of species and diversification rate, and means value of three traits, holoploid genome size (1C), monoploid genome size (1C*x*) and average DNA amount per chromosome (1C/n). P-values < 0.05 are printed in bold. For 𝜆, the mean value is given plus the minimum and maximum confidence value.**

| **Response** | **Predictor** | **λ (95% CI)** | **Slope** | **adj. *r^2^*** | **P-value** | **D.F.** |
| --- | --- | --- | --- | --- | --- | --- |
| total number of species | Mean 1C | 0.000 (NA, 0.475) | -0.29978 | -0.02497 | *P* = 0.5257 | 23 |
|  | Mean 1C*x* | 0.000 (NA, 0.445) | -0.47021 | 0.00000 | *P* = 0.3274 | 23 |
|  | Mean 1C/n | 0.000 (NA, 0.471) | -0.40667 | -0.01788 | *P* = 0.4547 | 23 |
| Diversification rate | Mean 1C | 0.371 (NA, 0.795) | -2.36172 | -0.00191 | *P* = 0.3388 | 23 |
|  | Mean 1C*x* | 0.393 (NA, 0.806) | -2.47952 | -0.00418 | *P* = 0.3526 | 23 |
|  | Mean 1C/n | 0.388 (NA, 0.801) | -2.95511 | 0.00000 | *P* = 0.3274 | 23 |

**
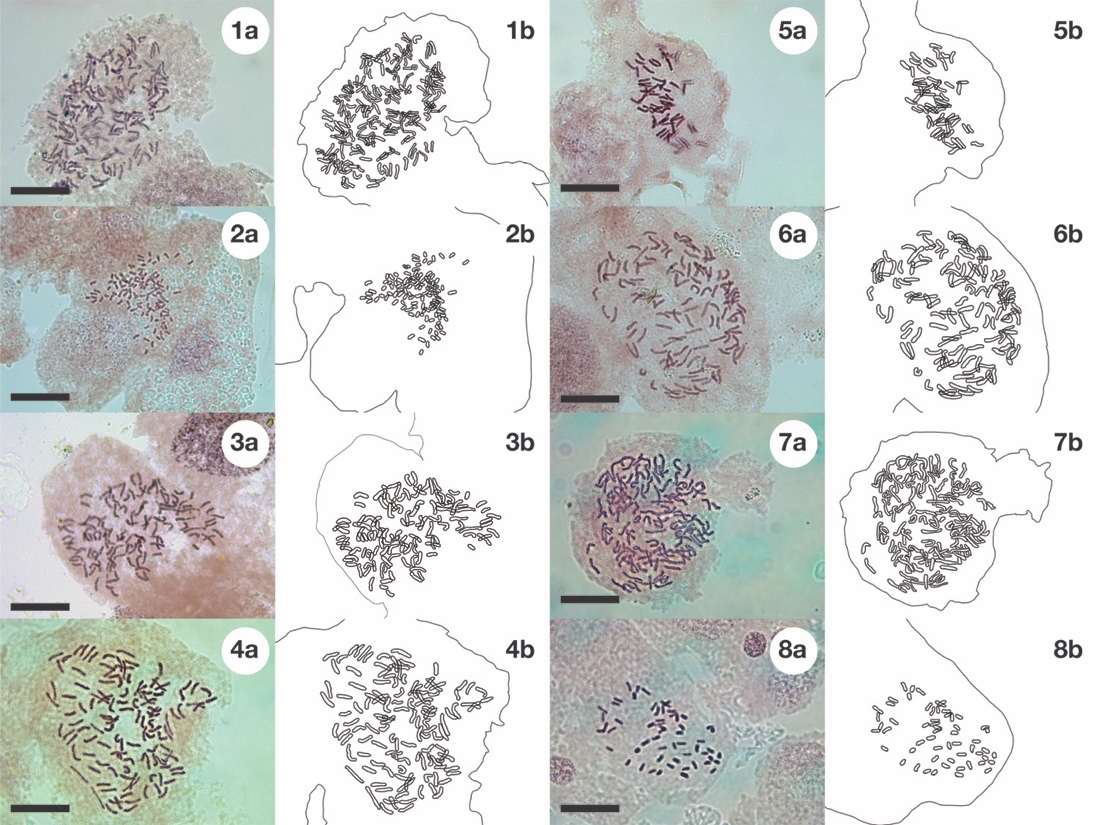
**

**Fig. S1 Mitotic metaphase chromosomes.** (a) microphotographs and (b) illustrations. Scale bars＝20 μm. 1. *Lindsaea javanensis* (2n = 188), 2. *Aleuritopteris subvillosa* (2n = 116). 3. *Cheilanthes tenuifolia* (2n = 116). 3. *Coniogramme emeiensis* (2n = 120). 5. *Coniogramme fraxinea* (2n = 60). 6. *Coniogramme sprapilosa* (2n = 120). 7. *Haplopteris forrestiana* (2n = 120). 8. *Pteris kawabatae* (2n = 58).

**
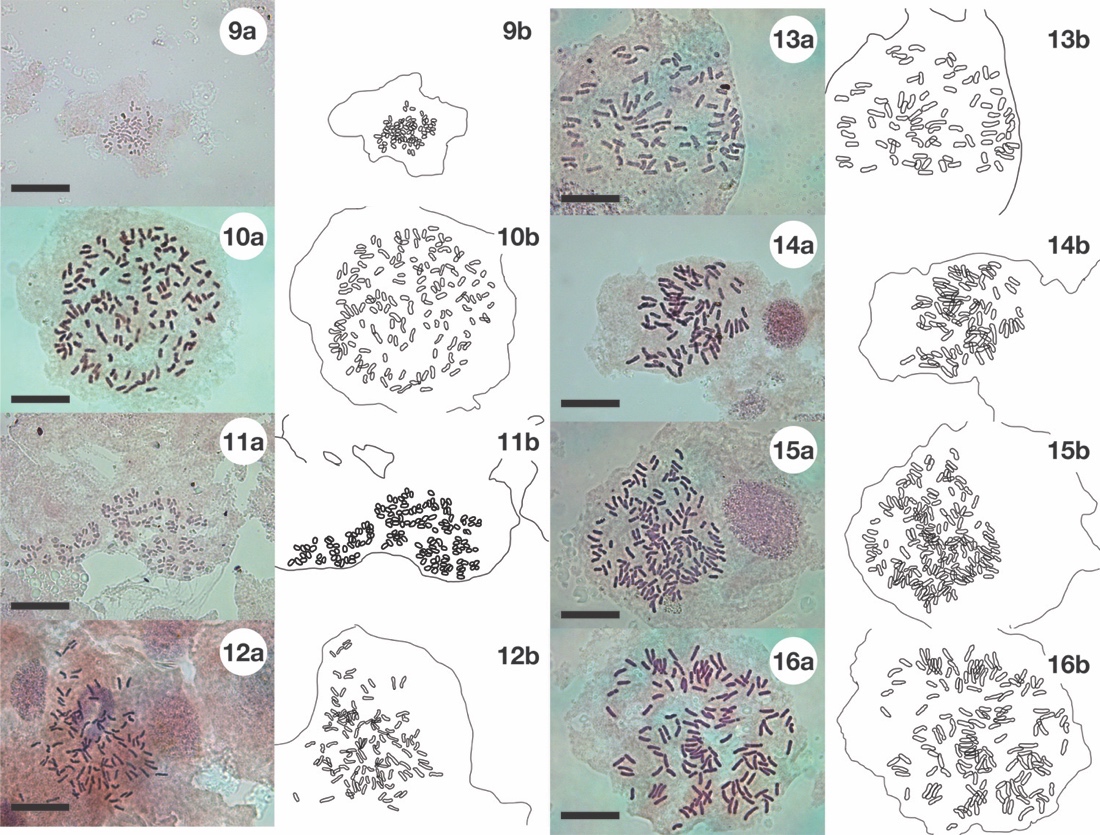
**

**Fig. S2 Mitotic metaphase chromosomes.** (a) microphotographs and (b) illustrations. Scale bars＝20 μm. 9. *Asplenium delavayi* (2n = 72). 10. *Asplenium pseudolaserpitiifolium* (2n = 144). 11. *Hypodematium crenatum* (2n = 164). 12. *Dryopteris stenolepis* (2n = c. 123). 13. *Bolbitis heteroclita* (2n = 82). 14. *Lomagramma matthewii* (2n = 64). 15. *Tectaria fauriei* (2n = 164). 16. *Lepisorus henryi* (2n = 144).

**
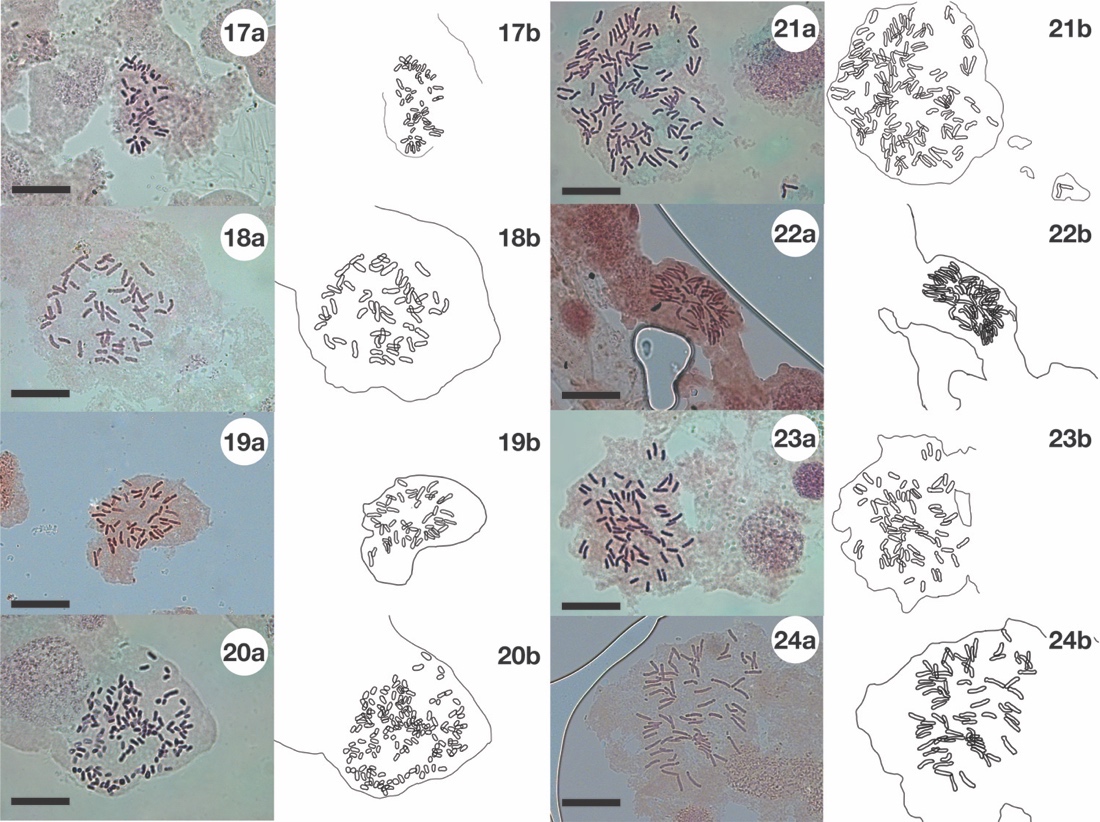
**

**Fig. S3 Mitotic metaphase chromosomes.** (a) microphotographs and (b) illustrations. Scale bars＝20 μm. 17. *Lepisorus obscurevenulosus* (2n = 50). 18. *Lepisorus pseudonudus* (2n = 52). 19. *Lepisorus sordidus* (2n = 50). 20. *Lepisorus* aff. *tosaensis* (2n = ca. 150). 21 *Leptochilus pteropus* (2n = 144).22. *Loxogramme cuspidata* (2n = ca. 70). 23. *Microsorum cuspidatum* (2n = 72). 24. *Microsorum insigne* (2n = 72).

**
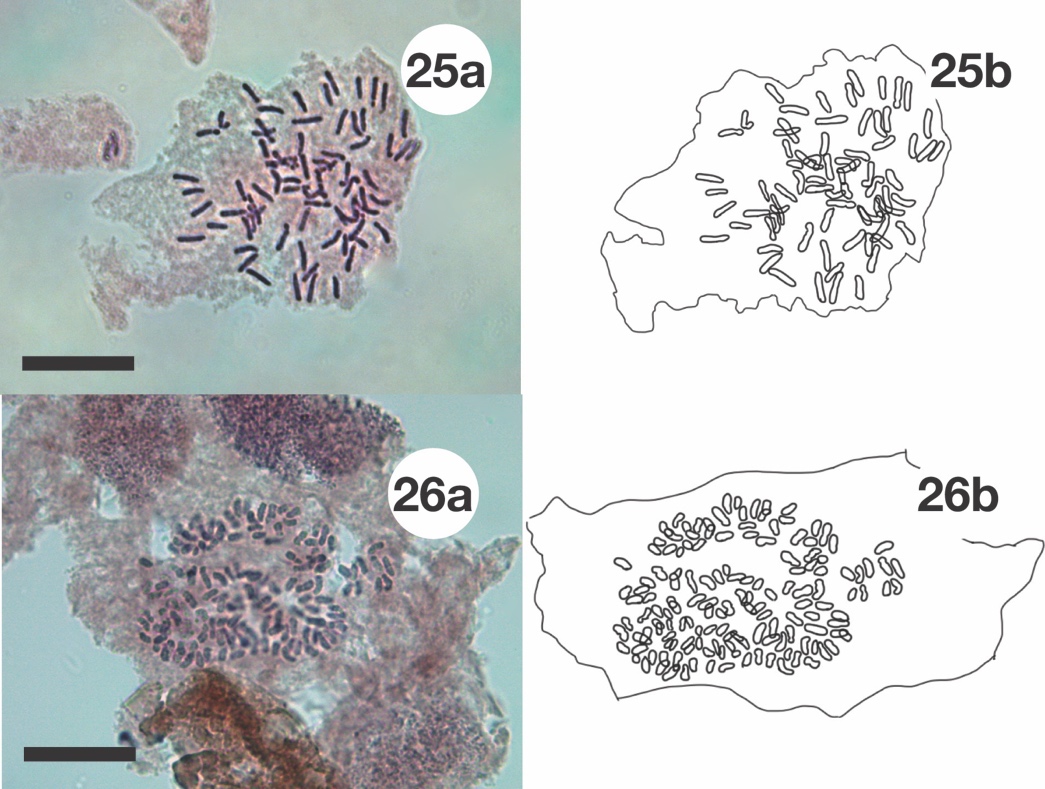
**

**Fig. S4 Mitotic metaphase chromosomes.** (a) microphotographs and (b) illustrations. Scale bars＝20 μm. 25. *Pyrrosia drakeana* (2n = 72). 26. *Pyrrosia tonkinensis* (2n = 144).

**Fig. S5 ML tree obtained in this study.** Different colored branches indicate different orders: Orange: Equisetales; Dark Blue: Psilotales; Pink: Ophioglossales; Light blue: Marattiales; Green: Osmundales; Light green: Hymenophyllales; Purple: Gleicheniales; Yellow: Schizaeales; Grey: Salviniales; Red: Cyatheales; Black: Polypodiales.

**Fig. S6 Ultrametric tree used in this study.** Different colored branches indicate different orders: Orange: Equisetales; Dark Blue: Psilotales; Pink: Ophioglossales; Light blue: Marattiales; Green: Osmundales; Light green: Hymenophyllales; Purple: Gleicheniales; Yellow: Schizaeales; Grey: Salviniales; Red: Cyatheales; Black: Polypodiales.

**Fig. S7 Phylogeny with clades painted to reflect rate shifts in Ornstein–Uhlenbeck parameters suggested by 1lou method for monoploid genome size (1C*x*).** Black squares and numbers indicate the locations of rate shift and bootstrap supports for these shift placements. Red and blue arrow near black squares indicates up and down shift in the rate shift. Bar plot located next to each phylogeny depicts trait value for each species and different colors show different orders. Classification according to PPGI.

**Fig. S8 Scatter plot showing the relationships between holoploid genome size (1C = y-axis) and average DNA amount per chromosome (n = x-axis) for All ferns”, “Homosporous ferns” , “Leptosporangiate ferns” and “Polypodiales”.** Each dot signifies one taxon. Each color corresponds to each order according to the right legend. dashed lines indicate the regression lines calculated using PGLS. Classification according to PPGI.

**Fig. S9 Scatter plot showing correlation between each of total number of species (A) and diversification rate (B), and mean values of three traits, holoploid genome size (1C) (left), the monoploid genome size (1Cx) (middle), and average DNA amount per chromosome (1C/n) (right).** Each dot signifies one taxon. Each color corresponds to each order according to the right legend. dashed lines indicate the regression lines calculated using PGLS. Classification according to PPGI.

**Fig. S10 Polypodiales phylogeny with clades painted to reflect rate shifts in Ornstein–Uhlenbeck parameters suggested by l1ou method for holoploid genome size, 1C (a) and average DNA amount per chromosome, 1C/n (b).** Black squares and numbers indicate the locations of rate shift and bootstrap supports for these shifts placements. Red and blue arrow near black squares indicates up and down shift in the rate shift. Bar plot located next to each phylogeny depicts trait value for each species and different colors show different orders. Classification according to PPGI.

**Supplemental References**

**PPGI**. **2016**. A community-derived classification for extant lycophytes and ferns. *Journal of Systematics and Evolution* **54**: 563–603.

**Regalado L, Schmidt AR, Krings M, Bechteler J, Schneider H, Heinrichs J**. **2018**. Fossil evidence of eupolypod ferns in the mid-Cretaceous of Myanmar. *Plant Systematics and Evolution* **304**.

**Regalado L, Schmidt AR, Müller P, Kobbert MJ, Schneider H, Heinrichs J**. **2017**. The first fossil of Lindsaeaceae (Polypodiales) from the Cretaceous amber forest of Myanmar. *Cretaceous Research* **72**: 8–12.

**Regalado L, Schmidt AR, Müller P, Niedermeier L, Krings M, Schneider H**. **2019**. *Heinrichsia cheilanthoides* gen. et sp. nov., a fossil fern in the family Pteridaceae (Polypodiales) from the Cretaceous amber forests of Myanmar. *Journal of Systematics and Evolution* **57**: 329–338.

**Schneider H, Schmidt AR, Heinrichs J**. **2016**. Burmese amber fossils bridge the gap in the Cretaceous record of polypod ferns. *Perspectives in Plant Ecology, Evolution and Systematics* **18**: 70–78.

**Skog JE, Banks HP**. **1973**. *Ibyka Amphikoma*, Gen. et sp. n., a New Protoarticulate Precursor from the Late Middle Devonian of New York State. *American Journal of Botany* **60**: 366.

**Testo W, Sundue M, Testo W, Sundue M**. **2016**. A 4000-species dataset provides new insight into the evolution of ferns A 4000-species dataset provides new insight into the evolution of ferns. *Molecular Phylogenetics and Evolution* **105**: 200–211.

**Wellman CH, Gensel PG, Taylor WA**. **2009**. Spore wall ultrastructure in the early lycopsid *Leclercqia* (Protolepidodendrales) from the Lower Devonian of North America: Evidence for a fundamental division in the lycopsids. *American Journal of Botany* **96**: 1849–1860.
